# Hygiene in Early Youth

**Published:** 1887-08

**Authors:** 


### Hygiene in Early Youth.

A three-year-old Philadelphian had heard her parents discuss hygiene until her infant mind was soaked with the subject. One day her dear old grandmother said—meaning to give Bessie a piece of cake—"Bessie, what do you always have after your bath?" The child regarded her grandmother for a moment with inquiring eyes, and then replied: "Reaction."—*Christian Intelligencer.*

"Pears to me," said old Uncle Pete, as he leaned his hoe against the corn-crib and abstracted a pebble from his shoe; "pears to me like dar was some kin' o' misdecomposishum in all dis talk adout babies cuttin' teef. De way I'se cum to look at it, hit's de teef cuttin' de baby. Leas'wise, dat's de way hit looks in de case ob cullud chilen."—*Archives of Pediatrics.*

